# Neurological Manifestations of COVID-19 (SARS-CoV-2): A Review

**DOI:** 10.3389/fneur.2020.00518

**Published:** 2020-05-22

**Authors:** Muhammad Umer Ahmed, Muhammad Hanif, Mukarram Jamat Ali, Muhammad Adnan Haider, Danish Kherani, Gul Muhammad Memon, Amin H. Karim, Abdul Sattar

**Affiliations:** ^1^Ziauddin University and Hospital, Ziauddin Medical College, Karachi, Pakistan; ^2^Khyber Medical College Peshawar, Hayatabad Medical Complex, Peshawar, Pakistan; ^3^Department of Internal Medicine, King Edward Medical University Lahore, Lahore, Pakistan; ^4^Allama Iqbal Medical College, Lahore, Pakistan; ^5^Houston Methodist Hospital, Houston, TX, United States; ^6^Liaquat National Hospital and Medical College, Karachi, Pakistan; ^7^Baylor College of Medicine, Houston, TX, United States; ^8^Southside Hospital Northwell Health, New York, NY, United States

**Keywords:** SARS-CoV-2, COVID-19, neurotropism, neurological manifestations, encephalitis, encephalomyelitis

## Abstract

**Background:** Coronavirus disease 2019 (COVID-19), caused by severe acute respiratory syndrome coronavirus 2 (SARS-CoV-2), has been associated with many neurological symptoms but there is a little evidence-based published material on the neurological manifestations of COVID-19. The purpose of this article is to review the spectrum of the various neurological manifestations and underlying associated pathophysiology in COVID-19 patients.

**Method:** We conducted a review of the various case reports and retrospective clinical studies published on the neurological manifestations, associated literature, and related pathophysiology of COVID-19 using PUBMED and subsequent proceedings. A total of 118 articles were thoroughly reviewed in order to highlight the plausible spectrum of neurological manifestations of COVID 19. Every article was either based on descriptive analysis, clinical scenarios, correspondence, and editorials emphasizing the neurological manifestations either directly or indirectly. We then tried to highlight the significant plausible manifestations and complications that could be related to the pandemic. With little known about the dynamics and the presentation spectrum of the virus apart from the respiratory symptoms, this area needs further consideration.

**Conclusion:** The neurological manifestations associated with COVID-19 such as Encephalitis, Meningitis, acute cerebrovascular disease, and Guillain Barré Syndrome (GBS) are of great concern. But in the presence of life-threatening abnormal vitals in severely ill COVID-19 patients, these are not usually underscored. There is a need to diagnose these manifestations at the earliest to limit long term sequelae. Much research is needed to explore the role of SARS-CoV-2 in causing these neurological manifestations by isolating it either from cerebrospinal fluid or brain tissues of the deceased on autopsy. We also recommend exploring the risk factors that lead to the development of these neurological manifestations.

## Introduction

The new public health pandemic COVID-19 is threatening the world with the outbreak of the novel corona virus (2019-nCOV) or severe acute respiratory syndrome corona virus 2 (SARS-CoV-2). In December 2019, a new virus epidemic in Wuhan, China (covid-19) (1) emerged as a world pandemic and has disseminated across many other countries (2). It has been declared a public health emergency by WHO. As of statistics obtained from Worldometer on April 11, 2020, the USA leads with over 0.5 million people infected, followed by Spain and Italy. The spread of this virus shows no evidence of plateauing and the economic, financial, social, and mental havoc along with severe lockdown measures is of great concern. The most common features reported have been shortness of breath, fever, and cough in the past since the epidemic but now new features, either as a result of sequelae or viral infection itself, are coming out. COVID-19 patients have been reported to develop many neurological symptoms ranging from headache to encephalitis (3). We present to outline the spectrum of different neurological manifestations in patients with COVID-19. Physicians should be cognizant of these manifestations while dealing COVID-19 patients.

## Microbiology, Origin, and Transmission

The coronavirus is an enveloped positive-sense single-stranded RNA virus belonging to the coronaviradae family. Under electron microscope, the virus appears crown-like due to the small bulbar viral spike (S) peplomers on the surface envelope. SARS-COV has shown to have a zoonotic origin with bats being the primary reservoir adapted by humans. It has shown to spread via respiratory droplets, fomites, and person-to-person contact. Transmission via stool shedding has also been established but has limited evidence (4).

## Pathophysiology

Many COVID-19 patients can develop neurological symptoms in addition to common respiratory symptoms as established by a retrospective case series study in Wuhan, China (3), which shows patients with severe COVID-19 develop more neurological symptoms such as acute cerebrovascular accidents, altered level of consciousness, and skeletal muscle damage as compared to those with mild infection. Li et al. proposed that the acute respiratory failure that occurs in COVID-19 patients could be partly because of the damage to the brain stem caused by SARS-CoV-2, in addition to direct damage to lungs (5). It raises a question over how SARS-CoV-2 enters brain? In this review article we present the possible mechanisms used by SARS-CoV-2 in causing the neurological presentations of COVID-19 by summarizing and recollecting different material published over time in this regard.

## Genome of SARS-CoV-2

SARS-CoV-2 is the seventh virus in the family of coronaviruses. Coronaviruses have positive-sense single-stranded RNA viruses in their genome and have spike membrane glycoprotein on their surface (6). Genetically SARS-CoV-2 is 79% identical to SARS-CoV and 50% to MERS-CoV (7). SARS-CoV and SARS-CoV-2 act via the angiotensin converting enzyme-2 (ACE2) as their main functional receptor (8), whereas MERS-CoV uses dipeptidyl peptidase 4 (DPP4 also known as CD26) as its predominant receptor. SARS-CoV and MERS-CoV cause many neurological manifestations in addition to respiratory symptoms (9, 10), as highlighted by the presence of the viral nucleic acid in the cerebrospinal fluid. This fact was later reinforced by the evidence of nucleic acid present in an autopsy of the brain (6).

Based on its structural homology with SARS-CoV and MERS-CoV, it can be stated safely that SARS-CoV-2 is neurotropic and there is a possibility that SARS-CoV-2 is also using the same mechanisms of pathogenicity for neurological manifestations (6).

## Mechanism of Targeting CNS

After a thorough search the mechanisms by which SARS-Cov-2 enters the CNS could be enunciated as follows:

1. Direct infection injury
2. Blood circulation pathway
3. Neuronal pathway
4. Immune mediated injury
5. Hypoxic injury
6. Other mechanisms.

## Direct Spread of SARS-CoV-2 From Cribriform Plate to Brain

One of the proposed mechanisms of SARS-CoV-2 entry into brain tissues is via dissemination and spread from the cribriform plate which is in close proximity to the olfactory bulb (11). This idea of direct spread could be supported by the presence of anosmia and hyposmia in COVID-19 patients as described by Mao et al. (3).

## Haematogenous Spread of SARS-CoV-2 to Target CNS

As previously mentioned, ACE 2 has been identified as the functional receptor for SARS-CoV-2 and varied expression and distribution of ACE2 receptors in different organs decide the severity of clinical manifestation of SARS-CoV-2 (12). ACE2 receptors are expressed on glial tissues, neurons, and brain vasculature which make them a target for the attack by SARS-CoV-2 (13). The role of blood-brain-barrier in preventing the virus entry is still to be established, but clinical manifestations of neurological symptoms in patients of SARS-CoV-2 in a recent study was established (3). This study included 214 patients, out of which 78 (36.4%) patients had some neurological symptoms, which strengthens our idea of the neurotropic potential of SARS-CoV-2 virus. Another case was reported that showed the presence of the virus in neuronal and vascular endothelial cells in frontal tissues detected on an autopsy of a confirmed COVID-19 patient (14).

S spike protein (encoded by mRNA) enables the binding of SARS-CoV-2 with ACE2 receptors in the same way as it does for SARS-CoV (15), and in a study it was seen that the binding affinity of SARS-CoV-2 protein S was 10 to 20-folds higher than SARS-CoV protein S (16). The presence of the virus in general circulation enables virus entry into cerebral circulation, where sluggish blood movement in micro vessels enables interaction of the viral spike protein with ACE2 receptors of capillaries endothelium (11). This subsequently leads to viral budding from capillary endothelium; resultant damage to the endothelial lining favors viral entry into the milieu of brain, where viral interaction with ACE2 receptors expressed over neurons can result in damage to the neurons without a substantial inflammation—seen previously with SARS-CoV infection (16). The avid binding of the virus to the ACE2 receptors can also result in their destruction via unknown mechanisms, leading to hemorrhage in the brain. Since ACE2 is a cardio-cerebral vascular protecting factor, its damage causes a leak of the virus in the CNS (13). It is important to mention that before the occurrence of anticipated neuronal damage with the virus, the endothelial damage in cerebral capillaries with resulting bleeding can have fatal consequences in COVID-19 patients.

## Neuronal Pathway

Another mechanism through which neurotropic viruses like the coronaviruses can reach CNS is by anterograde and retrograde transport with the help of motor proteins Kinesins and dynein via sensory and motor nerve endings (17), especially via afferent nerve endings of the vagus nerve from the lungs (5). In addition to this, SARS-CoV-2 can also cause gastrointestinal tract infection and can spread to the CNS via enteric nerve and sympathetic afferent (18). Moreover, Exosomal cellular transport is also a presumed pathway of SARS-CoV-2 systemic dissemination and subsequent CNS entry (19).

## Immune Mediated Injury to CNS

SARS-CoV-2 is proposed to cause damage to the Central Nervous System (CNS) by a surge of inflammatory cytokines (mainly Interleukin-6), called Cytokine Storm Syndrome (CSS), in the same way as many neurotropic viruses are assumed to induce the production of IL-6 from glial cells, resulting in cytokine storm syndrome (20). In an *in vitro* study, activated glial cells were seen to cause chronic inflammation and brain damage by producing pro inflammatory cytokines like IL-6, IL-2, IL-5, and TNFα (21). SARS-CoV-2 infection of CNS activates CD4+ cells of the immune system and CD4+ cells in turn induce the macrophage to secrete interleukin-6 (IL-6) by producing granulocyte-macrophage colony-stimulating factor. IL-6 is a predominant component of cytokine storm syndrome (CSS) and leads to multiple organ failure—a major cause of fatality in COVID-19 (22). This is further supported by the fact that treatment with Tocilizumab (IL-6 receptor blocker) resulted in improvement of critical ill COVID-19 patients (23). Based on the aforementioned fact, it is evident that cytokine storm syndrome is one of the many ways used by SARS-CoV-2 to damage the brain indirectly.

## Spectrum of Neurological Manifestations

Neurological manifestations of patients with COVID-19 are listed as below in the Table 1 (24) and Table 2.

**Table 1:** Spectrum of Neurological Manifestations of COVID-19.

Encephalitis
Anosmia/hyposmia
Viral meningitis
Post-infectious acute disseminated encephalomyelitis/Post-infectious brainstem encephalitis
Guillain Barré syndrome
Acute cerebrovascular disease

**Table 2:** Illustrating the Spectrum of Neurological Manifestations of COVID-19.

**Manifestations**	**Authors**	**Type of study**	**Presentations**	**References**
Encephalitis	Moriguchi et al. Poyiadji et al.	Case report Case report	Headache, fatigue, fever Fever, cough, altered mental status	(25, 26)
Anosmia	Gane et al. Eliezer et al. Klopfenstein et al.	Case report Case report Retrospective study	Hyposmia with headache and fatigue Isolated anosmia 54 out of 114 reported anosmia	(27–29)
Viral meningitis	Moriguchi et al. Duong et al.	Case report Case report	Headache, fatigue, fever Headache, fever, seizure	(25, 30)
Guillain–Barré syndrome	Zhao et al. Toscano et al. Sedaghat et al. Virani et al. Gutierrez-Ortiz et al.	Correspondence Case series Case report Case report Case report	Fatigue, leg weakness Four patients presented with lower limb paralysis and paresthesia and one presented with facial diplegia later on developed ataxia and paresthesia Quadriplegia after having cough, fever 2 weeks ago Bilateral numbness and weakness of lower limb extremities Diplopia after having diarrhea, fever and ageusia	(31–35)
Acute disseminated post-infectious encephalomyelitis/Acute disseminated post-infectious encephalitis	Arabi et al. Kim et al.	Retrospective study Case series study Note: (both studies are from Middle East Respiratory Syndrome)	Three patients presented with severe neurologic syndrome ranging from altered level of consciousness to coma, ataxia and focal motor deficit Four patients in cohort study presented with various neurological symptoms ranging from numbness to ataxia, ophthalmoplegia	(36, 37)
Acute cerebrovascular disease	Mao et al. Oxley et al. Avula et al. Al Saiegh et al.	Retrospective study Correspondence Case Series Case report	Five patients presented with ischemic stroke and one with hemorrhagic stroke Five patients presented with large-vessel stroke Four patients presented with CT proven stroke One patient presented with subarachnoid hemorrhage and the other had ischemic stroke with hemorrhagic conversion	(3, 38–40)

## Encephalitis

The most common underlying etiology of encephalitis or acute inflammation of the brain is viral infections like Herpes simples virus (HSV), Varicella zoster virus (VZV), cytomegalovirus (CMV), influenza virus (41), and many other respiratory viruses like severe acute respiratory virus coronavirus (SARS-CoV) and Middle East respiratory virus (MERS-CoV) (9, 10). As previously mentioned, SARS-CoV-2 can also have neurotropic effects because many COVID 19 patients present with neurological symptoms in addition to common respiratory symptoms (3). Moreover, recently the presence of SARS-CoV-2 RNA in the cerebrospinal fluid has been detected by genome sequencing in a patient with clinically proved meningoencephalitis in Japan (25).

Poyiadji et al. reported another case in which a female in her 50s presented with a 3 day history of fever, cough, and altered mental status and she was diagnosed with COVID-19 by detection of SARS-Cov-2 nucleic acid in a nasopharyngeal swab (26). Her cerebrospinal fluid analysis was negative for bacteria, HSV type 1 and 2, varicella zoster virus, and West Nile virus. A non-contrast head CT scan revealed symmetrical bilateral medial thalamic hypoattenuation with no abnormality seen on CT angiogram and CT venogram. Hemorrhagic ring enhancing lesions consistent with acute necrotizing encephalitis were seen in the bilateral thalami, medial temporal lobes, and sub-insular regions on an MRI (26). The above case report could support the potential idea that SARS-CoV-2 can cause encephalitis. Poyiadji et al. proposed that the virus does not directly invade the blood-brain-barrier and acute necrotizing encephalitis is caused by SARS-CoV-2 via cytokine storm.

Studies suggest that severe symptoms in COVID-19 patients might be due to cytokine storm syndrome (42). A cytokine profile characterized by increased IL-1, IL-2, IL-6, IL-7, tumor necrosis factor (TNF), macrophage inflammatory protein 1α, granulocyte colony stimulating factor, interferon-gamma inducible protein, and monocyte chemo-attractant protein is associated with the severity of COVID 19 (43). In clinical trials, the blocking of interleukin-1 receptor (IL-1R) with anakinra (44) and blocking of Interleukin-6 receptor (IL-6R) with tocilizumab (23) resulted in significant improvement in COVID-19 patients, which suggests that SARS-CoV-2-related damage is caused by cytokines.

## Anosmia

Anosmia means loss of the sense of smell and hyposmia means a reduced ability to smell. The most common neurological presentation of COVID-19 is anosmia/hyposmia, and in fact these can be the only presenting symptoms in a lot of patients, especially paucisymptomatic patients (45) as evident from the case report in which a patient presented with isolated sudden onset anosmia with no other symptoms of COVID-19 but tested positive for SARS-CoV-2 (27).

Eliezer et al. also reported a case in which a woman in her 40s presented with hyposmia with a history of dry cough along with headache and generalized fatigue a few days before presentation. The patient underwent testing for SARS-CoV-2 since she was in contact with her husband who was suspected to have COVID-19 and she was found to be positive (28).

A retrospective study among patients with anosmia by Klopfenstein et al. concluded that 47% (54 out of 114) of COVID 19 patients reported anosmia. Anosmia began 4.4 (±1.9 [1–8]) days after the onset of infection and the mean duration of anosmia was 8.9 (±6.3 [1–21]) days (29). Similarly, Lechien et al. found out that although the most prevalent symptoms of COVID-19 were cough, myalgia, and fever, olfactory and gustatory dysfunction was found in 85.6 and 88% patients, respectively, with significant association between the two disorders (*p* < 0.001). In 11.8% of the patients, olfactory symptoms appeared before other symptoms. Olfactory and gustatory dysfunction were more common in females as compare to males (*p* < 0.0001) which highlights a gender predisposition (46).

Anosmia is the most common neurological manifestation of SARS-CoV-2; strikingly it has been found mostly in patients in their early 20s and in otherwise asymptomatic and healthy patients (47). Reviewing the literature, we can conclude that every patient presenting with isolated anosmia should be screened for SARS-CoV-2, especially in this pandemic. To find out the exact mechanism on how SARS-CoV-2 causes anosmia, further research workup is needed (48).

## Viral Meningitis

Meningitis is the inflammation of the coverings of the brain and spinal cord. A case of SARS-CoV-2 related meningitis/encephalitis (25) has been reported in Japan, where a young patient presented with altered level of consciousness and a single episode of seizures (while he was being transferred to hospital). He had neck stiffness and his blood work up showed an increased white cell count and increased C-reactive proteins. A CT head showed no brain edema, but a CT chest showed small ground glass opacity on his right upper lobe and bilateral inferior lobes. Anti-HSV-1 and Varicella-zoster IgM antibodies were not detected in serum samples. An MRI performed later showed right lateral ventriculitis and encephalitis on his right mesial lobe and hippocampus. The MRI also showed pan-paranasal sinusitis. A RT-PCR test for SARS-CoV-2 detected SARS-CoV-2 RNA in the CSF but not in the nasopharyngeal swab. He was started on Laninamivir and antipyretic agents for headache, fever, and fatigue 9 days before admission. His chest ray and blood test were normal 5 days before admission. This case highlights the following:

1. SARS-CoV-2 is neuroinvasive.
2. We cannot exclude SARS-CoV-2 infection even if an RT-PCR for SARS-CoV-2 is negative on a patient's nasopharyngeal specimen.
3. SARS-CoV has been detected in the brain on autopsy by real time RT-PCR with a strong signal in the Hippocampus and in this patient inflammation was also found in the hippocampus; this reinforces the fact that SARS-CoV and SARS-CoV-2 share the ACE2 as a functional receptor.

A 41-year-old female with a known case of Diabetes Mellitus presented with headache, fever, and new onset seizures. She was awake, alert, and oriented to time, place, and person. She had neck stiffness and photophobia but no focal neurological deficit. Chest X-ray, computerized tomography (CT) head, liver function tests, renal function tests, electrocardiogram, and blood chemistry were normal. Cerebrospinal fluid analysis showed 70 white blood cells with all lymphocytes, 65 red blood cells, and 100 mg/dL proteins.

Ceftriaxone and Vancomycin, that were initially started, were stopped. Based on cerebrospinal fluid analysis she was diagnosed with a case of viral meningitis and was given Acyclovir. However, acyclovir was also discontinued upon negative polymerase-chain reaction for Herpes simplex virus (HSV).

She was treated with levetiracetam for seizures. She became disoriented, lethargic, confused, agitated, and had hallucinations. She was vitally stable. X-ray and computerized tomography (CT) chest were normal. Generalized slowing was observed on an Electroencephalogram without any epileptic discharges. SARS-CoV-2 testing ordered at the time of admission came back positive. She improved on hydroxychloroquine treatment. This case showed that COVID-19 patients can have only neurological symptoms at the time of initial presentation (30).

So, can SARS-CoV-2 cause meningitis? A plausible answer could be reassuring as mentioned by the case reports, but it needs further evaluation.

## Post-Infectious Acute Disseminated Encephalomyelitis/Post-Infectious Brainstem Encephalitis

Human corona viruses (HCoV-OC43) cause mild respiratory infections but sometimes many, like MERS-CoV, can cause severe neurological manifestations like acute disseminated post-infectious encephalomyelitis (36, 37) and post-infectious brain-stem encephalitis (49) because of their potential neurotropic traits (50). SARS-CoV-2, being a member of this family, can also result in these manifestations, especially in patients with autoimmune diseases like multiple sclerosis, myasthenia gravis, and sarcoidosis (24). But this is too early a stage for such manifestations of SARS-Cov-2 to be present and we need more research and investigation on it. Immunosuppressive therapies cause systemic immune suppression and could be of a great concern.

## Guillain Barré Syndrome

Guillain–Barré syndrome causes immune-mediated damage to the peripheral nerves that usually occur after gastrointestinal or respiratory illnesses. Most common antecedent infections are *Campylobacter jejuni* (51), Zika virus (52, 53), and influenza virus (54). Neuromuscular disorder has been reported with SARS-CoV by Tsai et al. (55) and similarly neurological manifestations like Bickerstaff's encephalitis overlapping with Guillain-Barré syndrome were also seen with MERS-CoV (37). As SARS-Cov-2 is very similar to SARS-Cov and MERS-Cov, it can also be an antecedent to Guillain–Barré syndrome.

SARS-CoV-2 may result in Guillain–Barré syndrome. There is a correspondence (31) published which mentions a 61-year-old woman presenting with acute leg weakness and fatigue and her blood work on admission showed Lymphocytopenia and Thrombocytopenia. She was diagnosed with Guillain–Barré syndrome on day 5 and was given Intravenous Immunoglobulin. On day 8 she developed a fever and dry cough and her oropharyngeal swabs were positive for SARS-CoV-2 on an RT-PCR assay. Her chest CT showed ground-glass opacities in both lungs.

They suspected that she might have been infected during her stay in Wuhan. Her abnormal laboratory findings on first presentation (Lymphocytopenia and Thrombocytopenia) could be because of SARS-CoV-2, as an early presentation of COVID-19 can be non-specific with fever only in 43.8% of patients on admission (56). This shows a parainfectious profile of association between GBS and SARS-CoV-2 as opposed to the classic post-infectious profile as reported in GBS associated with Zika virus (52, 53) and GBS associated with Influenza virus (54). She should have been tested on day 1 of presentation for SARS-CoV-2 to support our case more vigorously.

Five patients have been reported in Italy with Guillain–Barré syndrome after they had SARS-Cov-2 infection (32). Four patients had lower limb paralysis and paresthesia as the initial symptoms of Guillain–Barré syndrome while one patient initially had facial diplegia and later on developed ataxia and paresthesia. Three patients needed mechanical ventilation. The latency between the onset of COVID-19 symptoms and presentation of Guillain–Barré syndrome was from 5 to 10 days.

Nasopharyngeal swabs were positive for SARS-CoV-2 in four patients. One had a negative nasopharyngeal swab as well as negative bronchoalveolar lavage for SARS-CoV-2 but became serologically positive afterwards. Cerebrospinal fluid (CSF) analysis showed normal protein levels in two patients, white cell counts <5 in all five patients, and negative real-time polymerase-chain-reaction (RT-PCR) for SARS-CoV-2 in all patients. Electromyography showed fibrillation potentials initially only in three patients and later on in another. Magnetic resonance imaging with gadolinium showed caudal nerve roots enhancement in two patients and facial nerve enhancement in another patient, but no signal changes in two patients.

All four patients were treated with Intravenous immunoglobulin (IVIG); two were given a second course of IVIG and one underwent plasma exchange. After 4 weeks of therapy, two patients were still on ventilator support, two were having physiotherapy, and one was discharged as he was able to walk independently. All these reports showed a classical post-infectious profile of association between Guillain–Barré syndrome and other viruses as opposed to the above-mentioned case report (31).

Another case of association of Guillain–Barré syndrome with SARS-CoV-2 has been reported in Iran. A patient presented with quadriplegia and was admitted to hospital. Two weeks before he had a cough, fever, and dyspnea and had a positive reverse transcription polymerase chain reaction on oropharyngeal sampling. The patient had absent deep tendon reflexes with decreased vibration and fine touch sensation distal to ankle joint and bifacial nerve palsy. His brain magnetic resonance imaging (MRI) was normal but bilateral diffuse consolidation, ground glass opacities, and bilateral pleural effusion were seen on a CT chest. Electromyography findings were consistent with acute motor-sensory axonal neuropathy. The patient received intravenous immunoglobulin (33).

In another case, a 54-year-old patient presented with bilateral numbness and weakness of his lower extremities for 2 days. He also stated that he had a fever and dry cough for 10 days which did not improve with amoxicillin and steroids prescribed by his primary physician. Respiratory viral panel testing (Nasopharyngeal PCR) was sent and the test came out positive for Rhinovirus. A test result for SARS-CoV-2 was awaited. Magnetic resonance imaging (MRI) of his thoracic and lumber was done as the patient developed urinary retention. The MRI did not show any abnormality in the spine but showed bilateral basilar opacities in the lungs. Later on, the patient developed dyspnea and his weakness progressed up to his nipples and he was electively placed on a ventilator. Power in his lower extremities was 2/5 and 3/5 in his upper extremities. Deep tendon reflexes were absent. Guillain–Barré syndrome was diagnosed based on these findings and Intravenous immunoglobulin was given for 5 days (34). The patient's SAARS-CoV-2 test came back positive at two different testing facilities. Guillain–Barré syndrome in this case was thought to be post-SARS-CoV-2 infection as there was not any preceding respiratory tract infection or campylobacterial related diarrhea.

A variant of Guillain–Barré syndrome, Miller Fisher syndrome is also being reported to be associated with SARS-CoV-2 infection. A 50-year-old male with a 5 day history of fever, cough, malaise, headache, low back pain, and altered sensations of smell and taste presented with new onset double vision, perioral numbness, and ataxia. On examination, right internuclear ophthalmoparesis and right fascicular oculomotor palsy were noted. The patient tested positive for antibody GD1b-IgG. Real-time reverse-transcriptase—polymerase-chain-reaction done on an oropharyngeal swab was positive for SARS-CoV-2. Cerebrospinal fluid analysis, a computerized tomography of his head, and Chest X-ray were normal. The patient was labeled as having Miller Fisher syndrome. He was treated with intravenous immunoglobulin (35).

Another patient presented with diplopia 3 days after he had diarrhea, fever, and ageusia. Visual examination showed bilateral visual acuity of 20/25 and bilateral abducens palsy. Real-time reverse-transcriptase–polymerase-chain-reaction done on an oropharyngeal swab was positive for SARS-CoV-2. Cerebrospinal fluid analysis, a computerized tomography of his head, and Chest X-ray were normal. This patient had polyneuritis cranialis. He was treated with acetaminophen (35).

All these reports show that Guillain–Barré syndrome is associated with SARS-CoV-2 infection. We will have a better understanding of this association in the coming days as more cases pour in. However, clinicians should be very vigilant and take appropriate protective measures while dealing with such cases, especially in this era of COVID-19 pandemic as the patient can only have neurological findings at presentation and symptoms of COVID-19 may be overlooked, leading to the horizontal spread of the infection.

## Acute Cerebrovascular Disease

One of the many neurological manifestations associated with COVID-19, especially in those who suffer from a severe form of illness, is acute cerebrovascular disease. Mao et al. concluded that 5.7% of patients with severe COVID-19 developed acute cerebrovascular disease (3) and it usually presents as stroke, with ischemic strokes being more common than hemorrhagic strokes. SARS-CoV-2 infection associated with hypercoagulability is called “sepsis induced coagulopathy (SIC)” and the depletion of angiotensin-converting enzyme 2 (ACE2) results in tissue damage, including stroke (57). This was underscored by the fact that thrombolytic prophylaxis amongst critically ill ICU patients reduces the thrombotic complications with better outcome (58). As described earlier, avid binding of SARS-CoV-2 with ACE2 (a cardio-cerebro vascular factor) damages ACE2 and can lead to strokes (13). Moreover, cytokine storm syndrome associated with SARS-CoV-2 infection is also a potential cause of neuronal damage and stroke (22, 59).

Oxley et al. reported five patients who presented with large-vessel stoke. All of the five patients were under the age of 50 and four out of five patients had no previous history of cerebrovascular accidents. They all tested positive for COVID-19 and were diagnosed as COVID-19 related stroke after ruling out other potential causes (38). Similarly, in a case series, Avula et al. reported four patients who initially presented with computed tomography (CT) proven stroke and later tested positive for COVID-19 (39). These patients were screened to rule out other causes of strokes. Al Saiegh et al. reported two confirmed COVID-19 cases who presented with acute cerebrovascular disease. A young male without any previous history of hypertension or other chronic illness was diagnosed with acute subarachnoid hemorrhage possibly secondary to COVID-19 on head computed tomography (CT). This leads us to question whether subarachnoid hemorrhage could be a complication of COVID 19, but with limited data available more research is needed in this regard. The second patient, a 62-year-old female, had an ischemic stroke with hemorrhagic conversion on presentation without any heralding COVID-19 symptoms. She tested positive for COVID-19 later on. These two cases underscore the association of SARS-CoV-2 with cerebrovascular accidents (40). All the aforementioned cases illustrate that SARS-CoV-2 can lead to many cerebrovascular diseases directly or indirectly, however further studies are needed for validation.

Timely management plays a key role in determining the morbidity and mortality amongst patients with acute stroke. It is therefore needless to say that stroke teams and neurologists must be wary of the peculiar spectrum of the pandemic virus and devise appropriate strategies and personal protective measure at all circumstances. Further data is needed to establish knowledge about this condition.

## Conclusion

Neurological investigations and isolation of SARS-CoV-2 from cerebrospinal fluid indicate that SARS-CoV-2 is a neurotropic virus and causes multiple neurological manifestations.

Transcribrial spread of SARS-CoV-2 to the brain is supported by the fact that hyposmia/anosmia is one of the earliest symptoms with which patients usually present, but it needs to be further elucidated by isolating this virus from proximity to the olfactory bulb.

With a rapidly rising toll of COVID-19 patients with neurological manifestations, there is an urgent need to understand and diagnose the neurological symptoms earlier to prioritize patients and treatment protocols on the basis of the severity of the disease.

## Author Contributions

All authors have taken equal part in the study under mentorship of AK and AS.

## Conflict of Interest

The authors declare that the research was conducted in the absence of any commercial or financial relationships that could be construed as a potential conflict of interest.
